# Comparative analysis of connective tissue graft *versus* ozone-enriched platelet-rich fibrin in the treatment of Cairo class I gingival recessions: a randomized, double-blind, controlled clinical trial

**DOI:** 10.1590/1678-7765-2025-0565

**Published:** 2026-03-02

**Authors:** Marina Pereira Silva, Maria Ritha Veiga Colognese, Carlos Augusto Nassar, Veridiana Camilotti

**Affiliations:** 1 Universidade Estadual do Oeste do Paraná - UNIOESTE Faculdade de Odontologia Departamento de Dentística Restauradora Cascavel PR Brasil Universidade Estadual do Oeste do Paraná - UNIOESTE, Faculdade de Odontologia, Departamento de Dentística Restauradora, Cascavel, PR, Brasil; 2 Universidade Estadual do Oeste do Paraná - UNIOESTE Faculdade de Odontologia Departamento de Periodontia Cascavel PR Brasil Universidade Estadual do Oeste do Paraná - UNIOESTE, Faculdade de Odontologia, Departamento de Periodontia, Cascavel, PR, Brasil

**Keywords:** Connective tissue, Gingival recession, Platelet-rich fibrin, Oxygen, Ozone therapy

## Abstract

Gingival recession is characterized by the apical displacement of the marginal gingiva, exposing the root surface and causing aesthetic and functional issues such as dentin hypersensitivity and cervical lesions. Subepithelial connective tissue grafts (SCTG) are considered the gold standard for root coverage. Recently, ozone-enriched advanced platelet-rich fibrin (A-PRF) has emerged as a promising alternative due to its regenerative potential and biocompatibility. Objectives: To compare the clinical efficacy of ozone-enriched A-PRF and SCTG combined with the coronally advanced flap (CAF) technique to treat Cairo Class I gingival recessions. Methodology: This randomized, double-blind, controlled clinical trial included 22 healthy patients (mean age 32.23±11.86 years, range 20–60 years), each with bilateral Cairo Class I recessions. Sites were treated with either CAF + SCTG (control) or CAF + ozone-enriched A-PRF (test). Clinical parameters were assessed at baseline, 90, and 180 days. Data were analyzed using the Shapiro-Wilk, repeated measures ANOVA, Tukey, and Friedman tests (α=0.05) on GraphPad Prism 8.0®. This study adhered to the CONSORT guidelines for randomized clinical trials. Results: Complete root coverage (100%) was achieved in 13 of 22 sites (60%) in the SCTG group and in nine of 22 cases (43%) in the ozone-enriched A-PRF group. Both groups statistically and significantly improve over time in selected clinical parameters (p<0.05). The SCTG group showed significant gains in all evaluated outcomes, whereas the ozone-enriched A-PRF group showed significant improvements in clinical attachment level, recession height, recession width, and dentin hypersensitivity. SCTG more greatly increased gingival thickness (0.77±0.52 mm) and keratinized tissue width (0.81±0.95 mm) than ozone-enriched A-PRF (0.27±0.55 mm and 0.22±0.42 mm, respectively). Conclusions: Both treatment approaches effectively managed Cairo Class I gingival recessions. SCTG promoted superior soft tissue augmentation, whereas ozone-enriched A-PRF achieved comparable reductions in recession-related parameters. The limitations of this study include its short follow-up period and the absence of histological assessment. Clinical Trial Register: RBR-537bzqt

## Graphical abstract

**Figure:**
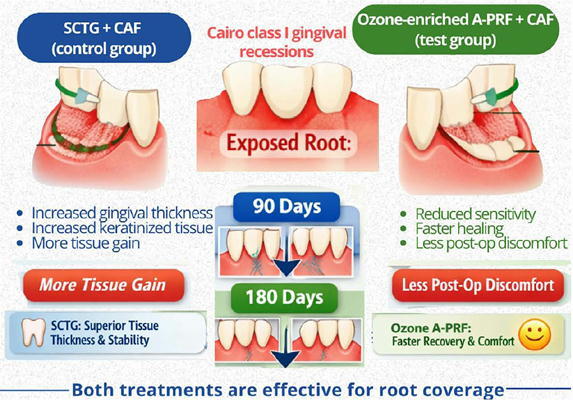

## Introduction

Gingival recession stands out as the most prevalent condition among mucogingival deformities, representing a major clinical concern because of its functional and aesthetic consequences. It is characterized by the apical migration of the gingival margin beyond the cementoenamel junction (CEJ), exposing the root surface.^1^ This exposure is frequently associated with dentin hypersensitivity, increased biofilm accumulation, root caries, non-carious cervical lesions, and compromised smile aesthetics.^2^,^3^ In this context, various surgical techniques have been developed to restore patient comfort and achieve optimal aesthetic outcomes.

The multifactorial etiology of gingival recession involves a complex interaction between anatomical, physiological, and behavioral factors. Among the most commonly reported are periodontal disease, occlusal trauma, traumatic toothbrushing, misaligned teeth, high frenal attachments, and muscle insertions that interact with the marginal gingiva.^4^ Other associated causes include chronic trauma, a thin gingival phenotype, and orthodontic tooth movement via a reduced buccal bone plate.^5^ Understanding these etiological factors is crucial for appropriate treatment planning and the prevention of further periodontal damage.

To standardize clinical assessment, Cairo, et al.^6^ (2011) proposed a classification system for gingival recessions based on interproximal attachment loss, according which Cairo Class I refers to gingival recessions without interproximal attachment loss, Cairo Class II involves interproximal attachment loss less than or equal to the buccal recession, and Cairo Class III is defined by interproximal attachment greater than the buccal recession. This classification can more consistently evaluate recession severity.

Over recent decades, periodontal surgical procedures have evolved to address these defects, focusing on achieving predictable root coverage.^7^, ^8^, ^9^ Among the numerous proposed surgical techniques, the coronally advanced flap (CAF) combined with a subepithelial connective tissue graft (SCTG) is considered the gold standard due to its superior outcomes in root coverage and enhancement of keratinized tissue. However, despite its high clinical success, this technique is associated with high postoperative morbidity, the need for a secondary donor site, risk of hemorrhage due to the greater palatine artery, and extended surgical duration.^10^, ^11^ These limitations have increased the interest in regenerative alternatives that reduce morbidity and maintain clinical efficacy.

In this context, platelet-rich fibrin (A-PRF) has emerged as an autologous biomaterial that can enhance soft tissue healing.^12^ PRF matrices are rich in leukocytes, cytokines, and growth factors that promote angiogenesis, modulate inflammation, and stimulate tissue regeneration.^13^ Several clinical studies have evaluated the use of PRF, particularly advanced PRF (A-PRF), in root coverage procedures. These investigations have reported satisfactory root coverage and improved soft tissue quality while offering the advantages of simplified preparation, cost-effectiveness, and elimination of the need for a second surgical site.^14^ Moreover, patients treated with PRF often experience lower postoperative morbidity and higher comfort than those who underwent SCTG-based procedures.^13^, ^14^, ^15^

Nonetheless, despite these benefits, the clinical predictability of PRF remains inferior to that of SCTG, particularly regarding gingival thickness gain, keratinized tissue width, and long-term stability.^16^ ^17^ ^18^ These limitations may be related to the faster degradation of the fibrin matrix and the variability in growth factor release among individuals.^19^ Therefore, strategies to enhance the biological performance and mechanical stability of PRF have gained increasing attention.

To potentially enhance the biological performance of A-PRF, recent research has explored its modification by incorporating medical ozone. Ozone therapy possesses antimicrobial, antioxidant, and immunomodulatory properties and has been applied in various dental specialties to support tissue healing.^20^ Anitua, et al.^16^ (2015) have investigated the ozonation of plasma-rich in growth factors, a preparation protocol distinct from A-PRF, and showed in *in vitro* studies that uncontrolled or high ozone doses can induce deleterious effects, such as impaired fibrin coagulation and degraded growth factors, whereas controlled doses showed no alteration of the properties of the biomaterial. More recent morphological observations by Schenato Delafiori, et al.^21^ (2025), using a protocol in which ozone was incorporated into the whole blood prior to centrifugation, have suggested that ozonated A-PRF shows a denser and more homogeneous fibrin network than untreated membranes. This structural improvement is hypothesized to enhance clot stability and clinical performance. However, the biological benefits of A-PRF ozonation remain largely speculative as direct evidence supporting its regenerative or clinical superiority remains lacking. For this reason, further preclinical and well-designed clinical studies are required to determine whether ozonation truly enhances the biological properties of A-PRF and to define safe and effective protocols for its clinical application.

Therefore, this randomized controlled clinical trial aims to compare the clinical effectiveness of ozone enriched A-PRF and SCTG combined with CAF to treat Cairo Class I gingival recessions. Its null hypothesis is that both techniques show no difference in root coverage or soft tissue stability, with the ozone-enriched approach achieving clinical equivalence while potentially offering greater patient comfort. The findings of this trial may provide valuable evidence to inform and refine clinical strategies in periodontal surgery.

## Methodology

### Study design

This randomized, prospective, double-blind, split-mouth controlled clinical trial was conducted. This design included patients presenting with bilateral Cairo Class I gingival recessions, allowing both interventions to be tested within the same individual and minimizing inter-individual variability. Each patient received a SCTG with a CAF + SCTG on one side (Control Group) and the CAF + ozone-enriched A-PRF on the contralateral side (Test Group). All surgical procedures were performed by a single calibrated operator. Postoperative assessments were conducted by a blinded examiner. The participants and the evaluator were unaware of the treatment allocation, thereby minimizing potential bias. This research was conducted at the Dental Clinics of Universidade Estadual do Oeste do Paraná in Cascavel, Paraná, Brazil.

### Ethical criteria

The experimental design in this research adhered to the CONSORT guidelines for randomized clinical trials. This study was officially registered in the Brazilian Registry of Clinical Trials under the identifier RBR-537bzqt (available at: https://ensaiosclinicos.gov.br/rg/RBR-537bzqt). The study protocol was reviewed and approved by the Research Ethics Committee of the State University of Western Paraná, Cascavel - PR, under the certificate number CAAE 71754823.6.0000.0107. All patients who met the inclusion criteria were thoroughly informed about the objectives, procedures, potential risks, and benefits of this study. Participation was strictly voluntary and formalized by participants signing of informed consent forms.

### Recruitment

The volunteers for this study were recruited via social media advertisements. They were initially screened by an examiner who was aware of the eligibility criteria. Individuals who met the inclusion criteria and showed no exclusion criteria were then invited to participate in the research. Each participant was informed verbally and in writing about the nature of this study, its procedures, and its potential risks and benefits related to the gingival recession treatment. Written informed consent was obtained from all participants prior to enrollment. The clinical procedures and data collection were conducted at the Dental Clinics of Universidade Estadual do Oeste do Paraná in Cascavel, Paraná, Brazil.

### Eligibility criteria

The patients in this study were required to have at least two Cairo Class I gingival recessions that were suitable for the split-mouth design. These recessions could be single or multiple and on homologous or non-homologous teeth within the same patient. Each patient contributed two recession sites, which were randomly assigned to receive either CAF + SCTG or CAF + ozone-enriched A-PRF, ensuring a split-mouth design to minimize inter-individual variability.

All teeth were required to be periodontally healthy, with probing depths ≤ 3 mm, bleeding on probing ≤ 5%, absence of gingival inflammation, and no carious lesions.

The following were considered as exclusion criteria: systemic diseases contraindicating surgery, current use of drugs that could influence periodontal status or healing (including antibiotics, steroidal, or non-steroidal anti-inflammatory drugs), smoking, pregnancy, lactation, occlusal interferences, carious lesions, previous mucogingival surgeries at the intended site, and Cairo Class II or III recessions. Also excluded were extruded, rotated, buccally displaced, or mobile teeth, and any presence of endodontic treatment or pulpal pathology.

Although sites included anterior and posterior teeth and maxillary and mandibular arches, no specific stratification by tooth type or arch was performed. These potential confounding factors were balanced by the random allocation of treatment sides and were considered during the interpretation of the clinical outcomes.

**Figure 1:**
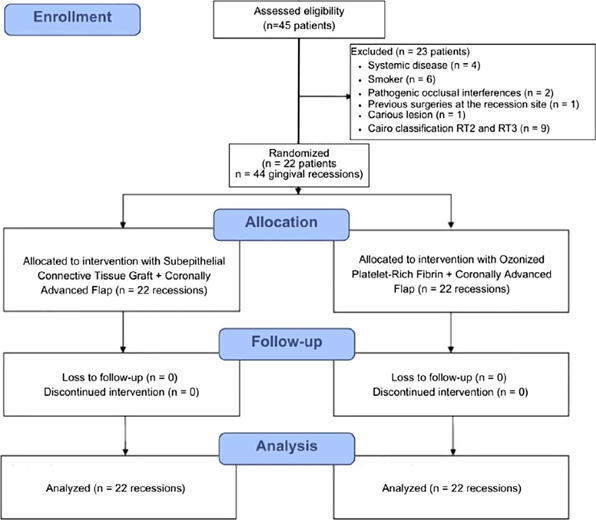
CONSORT flow diagram illustrating the allocation of interventions, follow-up, and analysis of the study groups.

The allocation of interventions, follow-up, and analysis of the study groups is shown in Figure 1 (CONSORT Flow Diagram).

### Sample calculation

Sample size was calculated based on the primary outcome. A power analysis for dependent samples (paired t-test) was performed with a significance level (alpha, α) = 0.05, statistical power (1-β) = 0.80, and effect size = 0.80. The minimum required sample size was determined to be 15 patients (30 recession sites). Effect size was estimated from the clinical differences in the prior split-mouth trials by Nassar, et al.^9^ (2014) and Bin, et al.^15^ (2023), with parameters of the recession height and clinical attachment level used as the reference for the sample size calculation. The analysis was performed on BioEstat 5.3 (Mamirauá Institute, Amazonas, Brazil).

### Randomization and allocation concealment

Simple randomization was performed using a web-based service (Sealed Envelope website: http://www.sealedenvelope.com) by an independent third party who had no involvement in the implementation or evaluation phases of this study. Given its split-mouth design, treatment allocation was determined by a site-specific draw, which selected the specific recession site to receive each intervention within the corresponding patient.

Allocation was concealed by opaque, sealed envelopes that were opened only at the time of intervention, preventing any prior knowledge of group assignment and minimizing selection bias.

Blinding was implemented for the patients and the evaluator to further minimize bias during this study. A second blinded examiner, responsible solely for the postoperative evaluations, performed all measurements without access to information about the used techniques or participants’ group allocation. Furthermore, patients were blinded to the type of graft applied to their recession sites. To ensure the integrity of blinding, all communications and records related to the specific used techniques were coded and securely stored by the principal investigator, making them inaccessible to the evaluator and participants.

### Team calibration

The team in this study underwent a rigorous calibration process to ensure the standardization of the assessments and surgical interventions. All surgeries were performed by a single experienced surgeon with a residency in oral and maxillofacial surgery, ensuring maximum uniformity in the execution of the techniques. Training began with an initial theoretical session, in which the study objectives, clinical protocols, and evaluation criteria for gingival recessions were described. This was followed by a practical training session, during which the operators practiced clinical examination techniques and procedures on simulated patients.

To ensure measurement consistency, the blinded examiner underwent intra-examiner agreement tests. The examiner assessed a set of previously selected gingival recessions at two time points, and the results were compared using an intraclass correlation coefficient. The examiner was only allowed to begin the study evaluations after achieving an intraclass correlation coefficient value of at least 0.8 (in which values closer to 1 indicated high concordance). Furthermore, calibration included the standardization of the use of clinical instruments (such as calibrated periodontal probes) and uniformity in the administration of the quality-of-life questionnaire.

### Surgical intervention

All surgical procedures were performed on the same day for each patient by a single experienced surgeon to ensure optimal technical standardization. The interventions included the Subepithelial Connective Tissue Graft (SCTG) combined with a Coronally Advanced Flap (CAF + SCTG) (Control Group) and the ozone-enriched Advanced Platelet-Rich Fibrin (CAF + ozone-enriched A-PRF) procedure (Test Group).

### Control group: subepithelial connective tissue graft with coronally advanced flap

#### Recipient site preparation

**Figure 2:**
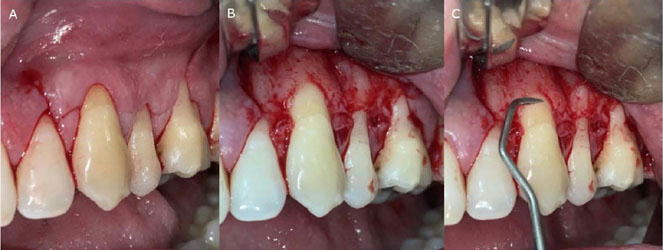
Surgical sequence for the coronally advanced flap with subepithelial connective tissue graft (SCTG). (a) Intrasulcular and vertical releasing incisions to outline the flap; (b) Full-thickness flap elevation beyond the mucogingival junction; (c) Recipient site preparation with root planing and papilla de-epithelialization.

Terminal infiltration anesthesia was administered using 2% Mepivacaine with Epinephrine 1:100,000 (DFL, Rio de Janeiro, RJ, Brazil). Site preparation began with an intrasulcular incision made with a 15-c blade (SOLIDOR®, Suzhou Kyuan Medical Apparatus Co. Ltd., China) followed by horizontal incisions at the base of the papillae at the CEJ and two vertical releasing incisions. A tension-free, full-thickness flap was then elevated apically beyond the mucogingival junction. Root surfaces were meticulously planed (or “debrided”) with a Gracey 7-8 periodontal curette (Hu-Friedy, USA), and the papillae were de-epithelialized with a 15c blade (Figure 2).

#### Donor site preparation

The SCTG was harvested from the palate under greater palatine nerve block anesthesia using the same anesthetic. A flap was designed using two horizontal and two vertical incisions made with a 15c blade. The necessary graft dimensions were confirmed using a calibrated periodontal probe. After a careful elevation of a partial-thickness flap (or a full-thickness flap followed by a partial-thickness split, depending on the technique), the SCTG was carefully excised (or “retrieved”). The donor site was subsequently closed with a 5-0 nylon suture (SHALON, Brazil).

#### Extraoral graft preparation and placement

**Figure 3:**
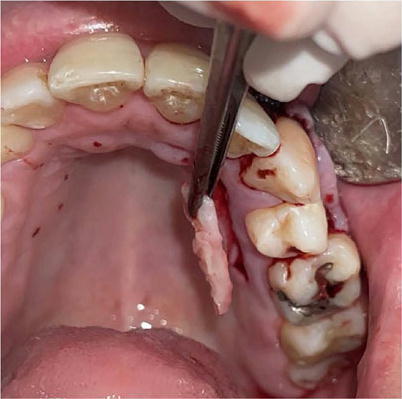
Graft removal from the palatal region following horizontal and vertical incisions.

The harvested graft was meticulously trimmed on a sterile field using a No. 11 blade (Swann-Morton, UK) to remove any residual epithelial tissue from the connective tissue (Figure 3). The prepared SCTG was then positioned directly over the denuded root surface, extending laterally beyond the recession margins. It was secured to the recipient site by 5-0 nylon sutures before the CAF was meticulously positioned and stabilized over it.

### Test group: Ozone-Enriched A-PRF with CAF

#### Blood collection and Ozonation Protocol

**Figure 4:**
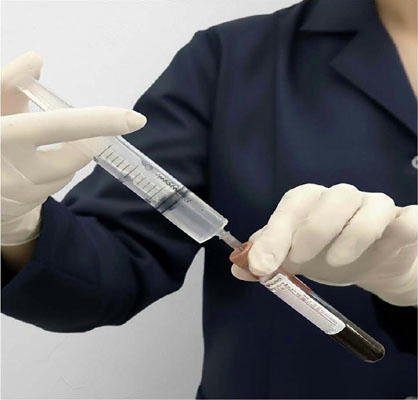
Incorporation of ozone gas into blood samples prior to centrifugation.

A total of 60 milliliters (mL) of venous blood was drawn into six 10 mL red-top clot activator tubes (Labor Import, Brazil). Ozone was generated at 10 μg/mL for a volume of 8 mL, corresponding to a 1:0.8 ratio (gas volume per blood volume). Immediately after collection, the oxygen-ozone gas mixture was injected directly into the tubes using a sterile syringe and needle (Figure 4). The samples were then gently agitated for 5 seconds to ensure homogeneous gas dispersion and optimal ozonation.

#### Membrane preparation

**Figure 5:**
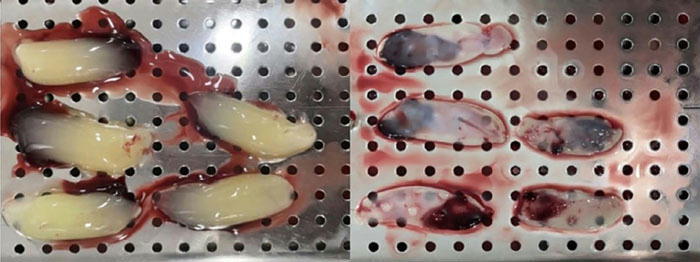
Final preparation of the membrane before placement at the recipient site. (a) Membrane placed on a perforated tray for controlled fluid drainage; (b) Gentle compression to standardize thickness and enhance tissue adaptation without compromising cell viability.

The ozonized blood samples were centrifuged (Kasvi K14-0815P) at 1300 RPM for 14 minutes. The resulting fibrin membranes were removed and placed on a perforated stainless-steel tray under a 130 g compression lid, ensuring gentle compaction without damaging the fibrin structure (Figure 5). The gaseous mixture of medicinal oxygen and ozone was prepared using the MedPlus MX generator (Philozon, Balneário Camboriú, Santa Catarina, Brazil), fed by a cylinder of pure medicinal oxygen. The oxygen was subjected to a controlled high-voltage electric discharge inside the device, partially converting it to ozone. The final concentration was adjusted on the digital panel of the generator and standardized to 10 μg/mL, corresponding to an appropriate clinical dose for this context. The resulting gas mixture was collected in a syringe immediately before application and applied to the fibrin membrane, ensuring the proper therapeutic concentration.

#### Placement in the Recipient Site

The prepared ozone-enriched A-PRF membrane was carefully positioned directly over the denuded root surface, covering the gingival recession defect. A single membrane was used in all cases. The membrane was stabilized at the recipient site using 5-0 nylon sutures, following a standard stabilization protocol. Subsequently, the CAF was meticulously advanced, positioned, and secured over the membrane using the same technique described for the Control Group.

#### Postoperative care and follow-up

All patients received a standardized postoperative protocol aimed at pain control, infection prevention, and inflammation management. The regimen included metamizole (dipyrone) 500 mg, one tablet every 6 hours for 3 days; amoxicillin 500 mg, one tablet every 8 hours for 7 days; nimesulide 100 mg, one tablet every 12 hours for 3 days; and mouth rinses with 0.12% chlorhexidine digluconate twice daily for 15 days. Surgical wound hygiene was performed using a cotton swab moistened with 0.12% chlorhexidine digluconate.

The sutures were removed at the donor site on the seventh postoperative day and at the recipient site on the 15th postoperative day. Clinical follow-up spanned 180 days, with evaluations conducted at baseline, 90 days, and 180 days. Throughout the study period, all participants were enrolled in a basic periodontal treatment and maintenance program (designed to eliminate etiological factors and prevent recurrence). The program included manual instrumentation with Gracey curettes 5–6, 7–8, 11–12, and 13–14 (Hu-Friedy, Chicago, IL, USA), occlusal adjustments when indicated, dietary counseling, and oral hygiene instructions. Patients were advised to avoid mechanical trauma to the surgical sites and to maintain meticulous plaque control.

#### Variables and clinical assessments

Data were systematically collected for all patients with gingival recession. This study analyzed several variables, including sex, age, involvement of the maxilla or mandible, localization in anterior or posterior teeth (with posterior teeth limited to premolars), and key clinical periodontal parameters such as recession height and width, probing depth, bleeding on probing, clinical attachment level, width and thickness of keratinized tissue, and the degree of dentin hypersensitivity. Overall, two groups were compared: the ozonized-A-PRF-with-CAF group and the SCTG-with-CAF group. Clinical assessments were conducted at baseline (T0), 90 days (T1), and 180 days (T2) following surgery to evaluate the progression and outcomes of the interventions.

**Figure 6:**
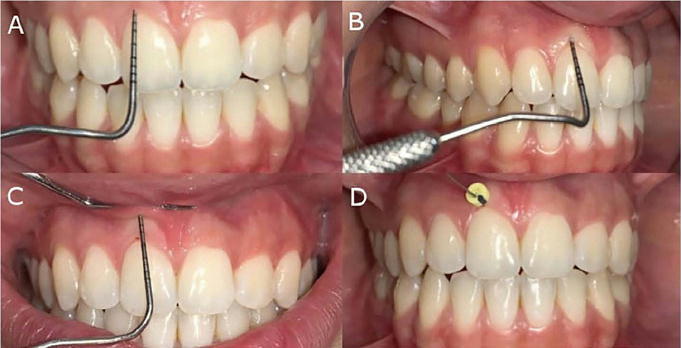
Clinical parameters used for outcome assessment. (a) Gingival recession height; (b) Probing depth; (c) Width of keratinized tissue; (d) Gingival thickness.

All clinical and periodontal measurements were performed by a single examiner who had undergone calibration. A Williams periodontal probe no. 23 was used for all evaluations. Recession height (RH) was defined as the distance from the CEJ to the gingival margin, measured in millimeters. Recession width (RW) was measured as the mesiodistal extent of the gingival margin. Probing depth represented the distance from the gingival margin to the base of the sulcus, whereas bleeding on probing was recorded as either present or absent following gentle probing. Clinical attachment level (CAL) was calculated as the sum of recession height and probing depth, representing the distance from the CEJ to the base of the sulcus. The width of keratinized tissue (KTW) was measured from the mucogingival junction to the gingival margin, whereas keratinized tissue thickness (KTT) was determined using an anesthetic needle to measure the distance from the epithelial surface to the buccal bone plate at the level of attached gingiva (Figure 6).

Dentin hypersensitivity (DHS) was evaluated using a Visual Analog Scale (VAS). It consisted of a continuous 10-cm line in which 0 indicated the absence of pain and 10 corresponded to the most severe pain. For this assessment, the stimulus was applied by directing a controlled air stream from a dental unit for three seconds directly over the gingival recession site. The adjacent teeth were meticulously isolated using cotton rolls to ensure the specificity of the stimulus to the test area.

### Statistical analysis

Data obtained from the 22 patients were systematically entered onto GraphPad Prism 8.0® for statistical evaluation. The normality of data distribution was assessed using the Shapiro-Wilk test. For intra-group comparisons across time points (T0, T1, and T2), parametric variables were analyzed using repeated measures ANOVA followed by Tukey’s post-hoc test for pairwise comparisons. Non-parametric variables, specifically pain and bleeding on probing, were analyzed using the Friedman test for repeated measures within each group. For inter-group comparisons of final mean values between the SCTG and ozone-enriched A-PRF groups, the paired Student’s t-test was applied. A significance level of 5% (p< .05) was adopted for all analyses.

## Results

**Figure 7:**
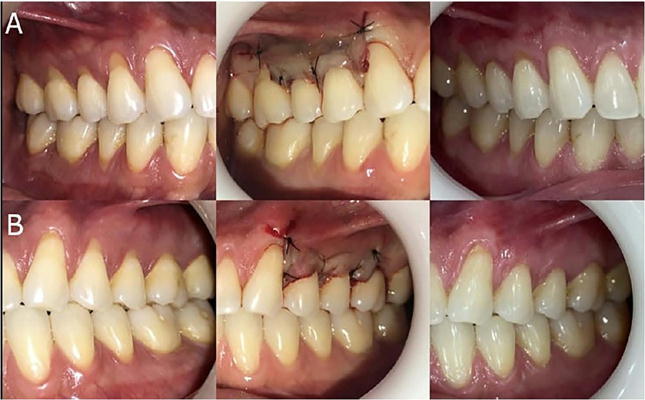
Clinical stages of the treatment groups. (a) Ozonized A-PRF + CAF group: Preoperative, intraoperative, and final follow-up views. (b) SCTG + CAF group: Preoperative, intraoperative, and final follow-up views.

A total of 44 gingival recessions were treated in 22 patients using a split-mouth design. These sites were equally allocated: 22 recessions to the SCTG + CAF group (Control) and 22 to the ozone-enriched A-PRF + CAF group (Test). Regarding demographics, the sample consisted of four men (18.2%) and 18 women (81.8%), with a mean age of 32.23±11.86 years. Age distribution by gender was unavailable due to the absence of gender-specific age records. Of the treated recessions, 14 were located on anterior teeth (31.82%) and 30 on posterior teeth (premolars) (68.18%), with 20 in the maxilla (45.45%) and 24 in the mandible (54.55%). Complete root coverage (100%) was observed in 13 (60%) of the SCTG + CAF group and nine (43%) of the ozonized A-PRF + CAF group. Clinical comparisons were conducted at baseline (T0), 90 days (T1), and 180 days (T2) in both groups (Figure 7).

Intra-group analysis across T0, T1, and T2 showed that both treatment modalities yielded statistically significant improvements (p<0.05) in all clinical parameters: CAL, KTW, RH, RW, KTT, and DHS, as in Table 1. These improvements remained throughout the 180-day follow-up. Specifically, the SCTG + CAF group showed significant gains in all six parameters (CAL, KTW, RH, RW, KTT, and DHS) from baseline to 180 days. In contrast, the ozone-enriched A-PRF + CAF group showed statistically significant improvements in CAL, RH, RW, and DHS. The comparative intra-group variation from baseline to 180 days for each periodontal parameter is shown in Table 2.

**Table 1:** Comparative periodontal parameters of SCTG + CAF and ozonized A-PRF + CAF at baseline, 90 days, and 180 days.

	Group 1 (SCTG + CAF)			Group 2 (ozonized A-PRF + CAF)		
	T0 (Baseline)	T1	T2	T0 (Baseline)	T1	T2
		(90 days)	(180 days)		(90 days)	(180 days)
**Probing depth**						
PD-M (mm)	1.95±0.95^A^	1.59±0.66^A^	1.63±0.72^A^	1.81±0.73^A^	1.54±0.59^A^	1.59±0.73^A^
PD-V (mm)	1.45±0.49^A^	1.27±0.63^A^	1.63±1.04^A^	1.54±0.50^A^	1.04±0.21^A^	1.09±0.29^A^
PD-D (mm)	1.72±0.70^A^	1.50±0.67^A^	1.72±0.93^A^	1.86±0.71^A^	1.31±0.47^A^	1.27±0.45^A^
**Bleeding and attachment**						
BOP	0.22±0.42^A^	0.18±0.39^A^	0.27±0.45^A^	0.36±0.49^A^	0.31±0.47^A^	0.22±0.42^A^
CAL (mm)	4.63±1.17^A^	2.63±1.64^B^	2.86±1.52^B^	4.59±1.56^A^	3.18±1.00^A^	2.90±1.15^B^
**Keratinized tissue and recession**						
KTW (mm)	3.72±0.93^A^	4.54±1.05^B^	4.54±1.05^B^	3.68±1.17^A^	3.90±0.97^A^	3.90±0.97^A^
RH (mm)	3.09±0.92^A^	1.36±1.36^B^	1.22±1.23^B^	3.18±1.40^A^	2.13±1.03^B^	1.77±1.19^B^
RW (mm)	3.54±0.91^A^	1.63±1.32^B^	1.59±1.33^B^	3.22±0.75^A^	2.54±0.50^B^	2.13±0.99^B^
KTT (mm)	1.36±0.49^A^	2.09±0.52^B^	2.13±0.56^B^	1.27±0.45^A^	1.59±0.50^A^	1.54±0.50^A^
**Postoperative pain assessment**						
DHS (VAS)	4.59±2.98^A^	2.00±2.42^B^	2.09±2.61^B^	5.09±2.67^A^	2.63±2.95^B^	2.54±2.93^B^

*Abbreviations: PD-M: Mesial probing depth; PD-V: Vestibular probing depth; PD-D: Distal probing depth; BOP: Bleeding on probing; CAL: Clinical attachment level; KTW: Keratinized tissue width; RH: Recession height; RW: Recession width; KTT: Gingival tissue thickness; DHS: Dentin hypersensitivity; VAS: Visual analog scale.

Different letters indicate statistically significant differences within each treatment group over time (p < 0.05).

**Table 2:** Comparative intra-group variation (baseline to 180 days) of periodontal parameters for SCTG + CAF and ozonized A-PRF + CAF.

Parameter	Group 1 (SCTG + CAF)	Group 2 (ozonized A-PRF + CAF)	^p^
PD-M (mm)	0.31±1.21^A^	0.22±0.92^A^	0.7807
PD-V (mm)	−0.18±1.09^A^	0.45±0.59^B^	0.0214
PD-D (mm)	0.00±0.87^A^	0.59±0.79^B^	0.0238
CAL (mm)	1.77±1.30^A^	1.68±1.32^A^	0.8198
KTW (mm)	0.81±0.95^A^	0.22±0.42^B^	0.0116
RH (mm)	1.86±0.88^A^	1.40±1.05^A^	0.1295
RW (mm)	1.95±1.32^A^	1.09±1.06^B^	0.0219
KTT (mm)	−0.77±0.52^A^	−0.27±0.55^B^	0.0037

*Abbreviations: PD-M: Mesial probing depth; PD-V: Vestibular probing depth; PD-D: Distal probing depth; CAL: Clinical attachment level; KTW: Keratinized tissue width; RH: Recession height; RW: Recession width; KTT: Keratinized tissue thickness. Different letters indicate statistically significant differences within each treatment group over time (p < 0.05).

Postoperative pain assessment using VAS showed that baseline and follow-up mean scores were higher in the ozonized A-PRF group than in the SCTG group. The mean VAS scores in the SCTG + CAF group decreased from 4.59±2.98 (T0) to 2.00±2.42 (T1) and 2.09±2.61 (T2). Similarly, in the ozonized A-PRF group, the scores decreased from 5.09±2.67 (T0) to 2.63±2.95 (T1) and 2.54±2.93 (T2). Despite both treatments reducing pain over time, the ozone-enriched A-PRF sites showed higher VAS scores across all time points, indicating lower postoperative comfort than in the SCTG group.

## Discussion

This randomized clinical trial compared the clinical performance of ozone-enriched A-PRF and SCTG associated with the CAF in the treatment of Cairo Class I gingival recessions. Both treatment modalities were effective in achieving root coverage and improving key clinical parameters, partially supporting the initial hypothesis of comparable clinical efficacy. However, the hypothesis of full clinical equivalence could not be entirely confirmed, as SCTG showed superior outcomes in gingival thickness, keratinized tissue width, and long-term stability. Moreover, the SCTG group showed greater postoperative comfort, indicating relatively more favorable patient-centered outcomes (PROMs) compared with the ozonized A-PRF group.

However, patient-reported morbidity and comfort are multifactorial. Clinical conditions that may modify these outcomes include baseline gingival phenotype, root and tooth position, extent of flap manipulation, and donor-site morbidity in SCTG.^19^, ^22^ Host-related factors such as age, systemic conditions, individual pain threshold, and local inflammatory response also modulate recovery.^19^, ^22^ Therefore, these factors should be considered when interpreting patient-centered outcomes and counseling patients.

The superiority of SCTG observed in this study is consistent with previous investigations showing that this technique remains the gold standard for root coverage procedures.^23^ Louis, et al.^18^ (2023) reported mean root coverage of 91% for SCTG and 86% for A-PRF. The percentage of complete root coverage (CRC) observed in the present study (60% for SCTG + CAF and 43% for ozonized-enriched A-PRF + CAF) was lower than that reported in some previous clinical trials, in which CAF combined with SCTG achieved rates ranging from 70% to 90% in Cairo Class I recessions.^1^ Several factors may account for this difference. First, site anatomical variations, such as a predominance of posterior teeth (68.18%) and mandibular sites (54.55%) in this sample, may have negatively influenced the surgical outcome, as root coverage is typically less predictable in these regions due to limited tissue thickness and muscle tension. Second, although a single calibrated surgeon performed all procedures, slight variations in flap tension or graft adaptation could have affected final coverage. Additionally, patient-related factors, including variations in gingival phenotype, oral hygiene practices, and individual healing responses, might have contributed to the observed variability. It should also be noted that the 180-day follow-up period may not be sufficient to capture long-term remodeling and creeping attachment phenomena, which can enhance root coverage outcomes over time. Despite these considerations, both techniques showed statistically significant clinical improvement in all key parameters, supporting their effectiveness in managing Cairo Class I recessions.

Research by Garzon, et al.^19^ (2021) and Mancini, et al.^23^ (2021) reported that SCTG provides greater root coverage and superior aesthetic outcomes compared to A-PRF. However, the authors also observed that A-PRF resulted in greater clinical attachment gain and reduced postoperative discomfort compared to SCTG. Similarly, in the present study, both groups showed improvement in recession height and width, but SCTG produced significantly thicker and more stable gingival tissue, which may represent a long-term clinical advantage.

Regarding gingival thickness and keratinized tissue width (KTW), the current results are consistent with the finding of Uzun, Ercan and Tunali^24^ (2018), that showed a significant increase in gingival thickness six and twelve months after SCTG procedures. Similarly, Miron, et al.^25^ (2020) confirmed that SCTG promotes a greater gain in keratinized tissue compared to A-PRF, corroborating the findings of our study. These improvements contribute not only to enhanced esthetics but also to better protection of the marginal periodontium and greater stability against future recession.

The incorporation of ozone into the A-PRF preparation aimed to enhance its biological properties by exploiting its known immunomodulatory, anti-inflammatory, and antimicrobial effects. The protocol adopted in our study involved the injection of ozone gas directly into the whole blood collection tube before centrifugation, similar to Schenato Delafiori, et al.^21^ (2025), a different approach from the plasma-based ozonation used by Anitua, et al.^16^ (2015). However, our method was strictly guided by the principle of controlled dosing established by Anitua, et al.^16^ (2015), whose *in vitro* work on plasma-rich in growth factors showed that excessive ozone concentration or uncontrolled flow (e.g., continuous flow) could induce deleterious effects, such as the degradation of growth factors and the impairment of fibrin architecture. Therefore, the use of a carefully controlled, low ozone concentration (10 μg/mL) in our protocol was paramount to maintain the structural integrity and biological potential of the A-PRF matrix. This caution is supported by the morphological findings of Schenato Delafiori, et al.^21^ (2025), who showed that ozonized A-PRF (prepared from ozonated blood) presents a denser and more homogeneous fibrin network, suggesting improved initial clot stability for tissue regeneration. While the current clinical results did not show superiority over SCTG, the favorable healing response and reduced postoperative discomfort observed in the ozonized A-PRF group suggest that the material is biologically active and clinically safe when prepared under this controlled protocol.

Despite these promising outcomes, SCTG remains the standard of care for gingival recession treatment due to its predictable and stable results in root coverage, tissue thickness, and keratinized tissue width.^13^, ^22^, ^24^ However, its limitations highlight the need for less invasive alternatives. In this context, ozonized A-PRF emerges as a potential substitute, particularly for patients contraindicated for graft harvesting or prioritizing comfort and faster recovery.^22^

The limitations of this study include the relatively short follow-up period of 180 days, which may not fully capture the long-term stability of root coverage and tissue remodeling. The absence of histological or molecular analyses also limits a deeper understanding of the biological effects of ozone incorporation into A-PRF. Furthermore, esthetic outcomes were not assessed, which represents a potential limitation. Future research should include larger sample sizes, longer follow-ups, and comparative histological analyses to confirm whether ozone-enriched A-PRF can truly enhance tissue regeneration and clinical predictability. Despite these limitations, the present findings provide valuable preliminary evidence supporting the clinical feasibility and biological potential of ozone-enriched A-PRF as an adjunctive approach in periodontal surgery.

Although ozonized A-PRF did not outperform SCTG in gingival augmentation or keratinized tissue width, it showed comparable improvements in recession reduction and significant advantages in postoperative comfort (a key PROM). These findings suggest that while SCTG remains superior in terms of tissue gain, ozone-enriched A-PRF offers clinically relevant benefits that align with the growing demand for minimally invasive, patient-centered treatments.

## Conclusion

Both treatment modalities (A-PRF and SCTG) efficaciously covered roots in patients with Cairo type I gingival recession over the follow-up period. SCTG showed superior outcomes regarding keratinized tissue width and gingival thickness, confirming its greater clinical performance than ozone-enriched A-PRF.

Ozone-enriched A-PRF provided notable benefits in reducing postoperative morbidity and improving patient comfort and outcomes, making it a viable alternative in cases in which SCTG is contraindicated or when minimizing surgical discomfort is prioritized. Both treatment approaches maintained clinical stability throughout the evaluated periods (baseline, 90 days, and 180 days), suggesting consistent and durable outcomes.

Overall, ozone-enriched A-PRF may be recommended for patients seeking a less invasive treatment option with a lower risk of postoperative complications. Conversely, SCTG remains the preferred choice for those aiming to achieve optimal gains in gingival thickness and keratinized tissue width. These findings highlight the importance of individualized treatment planning based on patient needs and clinical objectives.

## Data Availability

All data generated and analyzed in this study are included in this published article.
